# Applying behavioral change theories to optimize pulmonary rehabilitation in COPD patients: A review

**DOI:** 10.1097/MD.0000000000038366

**Published:** 2024-05-31

**Authors:** Yuyin Chen, Ruyi Tan, Xiuhong Long, Huiqiong Tu

**Affiliations:** a School of Nursing, Guangxi University of Chinese Medicine, Nanning, China; b Nursing Department, Ruikang Hospital Affiliated to Guangxi University of Chinese Medicine, Nanning, China.

**Keywords:** chronic obstructive pulmonary disease, health behavior change, pulmonary rehabilitation, theoretical models

## Abstract

This review meticulously evaluates the integration of behavioral change theories into pulmonary rehabilitation programs for chronic obstructive pulmonary disease (COPD) management, addressing the critical need for enhanced patient compliance and improved therapeutic outcomes. With COPD posing significant global health challenges, characterized by high morbidity and mortality rates, the manuscript underscores the potential of Self-Determination Theory, Social Cognitive Theory, the Transtheoretical Model, the Health Belief Model, and the Theory of Planned Behavior to foster meaningful health behavior changes among patients. Through a comprehensive literature analysis, it reveals how each model contributes to understanding patient behaviors in pulmonary rehabilitation contexts, advocating for their systematic application to craft more effective, patient-centered interventions. Despite the proven efficacy of these theories in various health domains, their current underutilization in pulmonary rehabilitation underscores a gap between theoretical knowledge and clinical practice. The review calls for an interdisciplinary approach that bridges this gap, highlighting the urgency of developing actionable, theory-based behavioral intervention plans. By doing so, it aims to advance COPD management strategies, ultimately improving the quality of life for individuals living with this debilitating disease.

## 1. Introduction

Chronic obstructive pulmonary disease (COPD), a chronic respiratory condition that is both preventable and manageable, is marked by persistent respiratory symptoms resulting from anomalies in the airways and/or alveoli, leading to airflow limitation.^[1]^ With the progression of COPD, individuals may also encounter severe comorbidities including pulmonary arterial hypertension, chronic cor pulmonale, and respiratory failure, significantly diminishing life quality and exacerbating the burden of illness.^[2,3]^ Current surveys reveal a global COPD prevalence of approximately 212.3 million cases, contributing to over 3.3 million fatalities annually.^[4]^ The World Health Organization anticipates a steady increase in COPD incidence, forecasting more than 5.4 million deaths annually by 2060 due to the disease and its related complications.^[5]^ Notably, in China, the COPD case count nears 10 million, with death tolls projected to ascend to 1.054 million by the year 2030.^[6]^ Given its extensive prevalence, high mortality, and significant impact on disability, COPD has emerged as a formidable public health challenge across the globe.^[7]^

Currently, the management of COPD predominantly centers on pharmacological treatments, including bronchodilators, inhaled corticosteroids, mucolytics, and immunomodulators, which aim to control symptoms during acute exacerbations and prevent their recurrence.^[8]^ Given the chronic and recurrent nature of COPD, effective disease management requires more than just hospital treatment during acute exacerbations; it necessitates a comprehensive approach that includes systematic and scientific rehabilitation management during the periods of remission. Recent research underscores the growing prominence of pulmonary rehabilitation as a critical component in the prevention and treatment of COPD.^[9]^

Pulmonary rehabilitation represents a comprehensive and personalized intervention strategy, forming the cornerstone of COPD management. Recognized as an effective nonpharmacological therapy,^[9]^ it significantly alleviates respiratory symptoms, enhances physical fitness, and improves quality of life, establishing itself as one of the most cost-effective treatments for COPD.^[10]^ Its key components include exercise training, respiratory muscle training, nutritional support, health education, and psychological interventions. Reports published by the American Thoracic Society and the European Respiratory Society ^[11–13]^ have underscored the critical importance of pulmonary rehabilitation. In terms of fostering behaviors conducive to pulmonary rehabilitation, current interventions such as health education,^[14]^ home-based rehabilitation,^[15]^ and telemedicine^[16,17]^ have shown remarkable efficacy. Despite substantial progress, there remains a need for integration among pulmonary rehabilitation program schemes, health behavior transformation, and theories of behavioral change.

Scientific application of behavior change theories can enhance patient compliance with pulmonary rehabilitation programs, thereby improving the outcomes of such interventions. However, currently, only a minority of researchers utilize these theories to guide their studies, resulting in inadequate application of these theories to facilitate patients in altering their health behaviors and enhancing self-management capabilities.^[18,19]^ Consequently, there is an urgent need for robust theoretical support to develop practical and actionable pulmonary rehabilitation behavioral intervention plans and measures. In light of this, the present study aims to review 5 established behavior change theories, delving into the essence of each and their current application status, strengths, and limitations in the context of pulmonary rehabilitation health behavior research. This endeavor seeks to heighten healthcare professionals’ awareness of relevant theories while providing a reference for selecting the appropriate theoretical model.

## 2. Current application of theoretical models in pulmonary rehabilitation health behavior

At present, the theoretical models employed in pulmonary rehabilitation health behavior research primarily focus on altering patients’ health-related behaviors to foster positive rehabilitation outcomes. This paper highlights several key theories: Self-Determination Theory (SDT), Social Cognitive Theory (SCT), the Transtheoretical Model (TTM), the Health Belief Model (HBM), and the Theory of Planned Behavior (TPB).

### 2.1. Self-determination theory

Introduced by Deci and Ryan in 1985, SDT has evolved over nearly 3 decades into a leading paradigm. It has distinguished itself in the field of positive psychology through a series of continuous theoretical expansions, innovations, and a wealth of empirical research. SDT articulates the impact of external factors, such as the environment and objects, on individual behavior through internal psychological processes, highlighting the influence of intrinsic and extrinsic motivation.^[20]^ The theory emphasizes the importance of individuals’ self-determination in the behavioral process and the active role in the motivational process. It offers a way to understand individuals’ motivational behaviors and personality formation, representing intentional behavior. It focuses on how individuals naturally develop and change over time, regulating their behavior, thereby suggesting that interventions based on SDT can effect long-term behavioral changes, such as in pulmonary rehabilitation health behavior interventions. Over the years, the theory has expanded to include 5 mini-theories: Basic Psychological Needs Theory, Cognitive Evaluation Theory, Causality Orientations Theory, Goal Contents Theory, and Organismic Integration Theory.^[21]^

In recent years, SDT has garnered attention in the health behavior field, with a significant body of research evidence supporting its impact. Ntoumanis et al^[22]^ included 73 related publications in their meta-analysis, which revealed that interventions based on SDT led to mild to moderate changes in health behaviors, along with positive physical and psychological health outcomes. Specifically in the realm of pulmonary rehabilitation health behavior, Drennan and Ross^[23]^ included 33 COPD patients, divided into an intervention group of 15 and a control group of 18. The intervention group participated in an SDT-based motivational interviewing pulmonary rehabilitation program. After undergoing 11 sessions over a period of 10 weeks, significant differences were observed between the intervention and control groups in terms of basic psychological needs, exercise-induced breathlessness, and functional status. Furthermore, Knox et al^[24]^ conducted a cross-sectional survey using SDT, Health-Related Quality of Life, and self-management knowledge questionnaires on 72 moderate to severe COPD patients who had not participated in pulmonary rehabilitation. The results indicated that SDT concepts could be applied to predict controlled and autonomous regulation of self-management, self-management knowledge, and Health-Related Quality of Life in moderate to severe COPD patients who had not participated in pulmonary rehabilitation. This insight could inform targeted interventions to promote patient self-management behaviors, effectively increasing participation in pulmonary rehabilitation.

In summary, SDT is currently the most thoroughly researched and widely applied motivation theory based on humanistic principles. This theory recognizes that individuals make free choices about their actions based on a comprehensive understanding of personal needs and environmental information. SDT acknowledges the humanistic view that humans are inherently proactive in seeking self-development, possessing an intrinsic tendency toward self-integration that propels individual growth and self-actualization. It affirms the innate proactive nature of humans and emphasizes the importance of a supportive social environment. Only when an individual’s basic psychological needs are met can their full proactive potential be realized.

### 2.2. Social cognitive theory

This theory was proposed by Bandura,^[25]^ who posited that learning occurs through observation, and social learning results from a dynamic interaction between an individual’s cognitive thought and their surrounding environment. SCT highlights the unique ways individuals acquire and maintain behavior, while also considering an individual’s past experiences and the social context in which behavior is enacted. These factors are believed to influence an individual’s eventual actions.^[26]^ Central to SCT are the concepts of triadic reciprocal determinism, self-efficacy, and observational learning. The core concept, triadic reciprocal determinism, posits a causal relationship among behavior, personal thoughts, and the social environment, where each influences and is influenced by the others. Self-efficacy, a critical concept within SCT, plays a pivotal role in health behavior. It refers to an individual’s belief or confidence in their capability to successfully execute specific behaviors and control influencing factors. Observational learning suggests that individuals learn by observing the behaviors of others, thereby forming their own cognitions.^[27]^

Jolly et al^[28]^ recruited 577 COPD patients and randomly assigned them to either an intervention group (n = 289) or a control group (n = 288). The intervention group received SCT-based telephone health coaching, while the control group received standard care. Postintervention results indicated that, compared to the standard care group, the intervention group reported higher levels of physical activity over 6 months of follow-up, a greater proportion adopted the care plan (44% vs 30%), accepted antibiotic rescue packs (37% vs 29%), and underwent inhaler technique checks (68% vs 55%). Aliakbari et al^[29]^ randomly divided 70 COPD patients into an experimental group and a control group, with the former receiving 4 SCT-based educational training sessions, including lectures, group discussions, and Q&A sessions, while the latter received conventional measures. The results showed that SCT-based interventions empowered patients, increasing daily activities among COPD sufferers. Kosteli et al^[30]^ interviewed 26 COPD patients from an SCT perspective to identify factors that promote or hinder physical activity participation among them. Findings underscored the significance of self-efficacy in effecting health behavior change and suggested that patient compliance with physical activities could be enhanced by building participants’ confidence. Additionally, patients’ positive outcome expectations regarding physical activities also influenced their participation behavior.

In conclusion, SCT has been applied in many areas, effectively incorporating research findings from humanistic psychology and cognitive psychology. SCT provides an explanation of human behavior from the perspective of agency, suggesting that individual factors, environmental events, and behaviors interact with each other. It occupies a central position in the field of health behavior, often used to understand and explain predictions and analyses regarding individual behavioral tendencies. The aim is to offer insights for a broad understanding of behavioral changes in individuals or groups.

### 2.3. Transtheoretical model

The TTM also known as the Stages of Change Model, was proposed by American psychologist Prochaska.^[31]^ It emerged from the integration of various mainstream theoretical structures in the fields of psychotherapy and behavior change. TTM posits that before selecting the appropriate behavior change strategies for an individual, it is essential first to understand the stage of behavior change in which the individual is situated. This theory highlights that individual behavior represents a dynamic, evolving, and stage-based process rather than a static one. It advocates for the adoption of different behavior change plans at different stages, tailored to the individual’s specific psychological and behavioral change patterns. The aim is to gradually transition or establish healthy behaviors until the health goals are achieved.^[32]^ TTM encompasses 5 stages: precontemplation, contemplation, preparation, action, and maintenance.

Chen et al^[33]^ recruited 98 COPD patients and randomly assigned them to either an experimental group or a control group. The experimental group underwent a pulmonary rehabilitation exercise intervention based on the TTM, whereas the control group received standard pulmonary rehabilitation exercises. The findings postintervention indicated that the TTM-based pulmonary rehabilitation exercise intervention effectively enhanced COPD patients’ adherence to rehabilitation exercise behaviors, improved their quality of life, and increased lung function. In a related study, Hellem et al^[34]^ employed TTM in their qualitative research to explore COPD patients’ perceptions regarding the critical factors for maintaining an exercise regimen. Their approach emphasized healthful and beneficial changes over the causes of the disease, recognizing the strengths and adaptability of the patients.

In summary, the TTM views behavior as a process rather than as a static, unchanging event. It emphasizes progression from 1 stage to another, accounting for individuals at different stages of behavioral change. This not only allows patients to clearly perceive the benefits of adopting healthy behaviors but also enhances their engagement, thereby improving health outcomes. However, the division into 5 stages of change is subject to subjective assessment by researchers, which can lead to misunderstandings and impact study results. Future efforts should focus on continuously applying and refining this cross-theoretical model through practical evaluation in patient behavior change initiatives.

### 2.4. Health belief model

The HBM was initially proposed in 1958, with Rosenstock et al^[35]^ further developing and refining it to identify the reasons and factors influencing individuals’ failure to engage in preventive health behaviors. This theory incorporates several variables, including individuals’ perceptions of the threat posed by health issues (perceived susceptibility and perceived severity), the benefits of avoiding these threats (perceived benefits), and factors influencing decision-making processes regarding behaviors (perceived barriers, cues to action, and self-efficacy).^[36]^ HBM posits that health beliefs are a prerequisite for the effectiveness of health behaviors. When individuals recognize the severity of adverse outcomes, perceive threats to their health from their environment, and believe that taking health-related actions is effective and feasible with minimal cost or effort, they become confident in their ability to achieve their goals. Consequently, individuals are likely to adopt health behaviors, thereby altering previously harmful behaviors and fostering a healthy lifestyle.^[37]^

Simon^[38]^ conducted a study to assess the attitudes of Hungarian asthma and COPD patients toward treatment adherence. Utilizing the HBM as a theoretical framework, the researchers surveyed 147 COPD patients. The results indicated that over 32% of patients were highly interested in new research findings on asthma or COPD, but their primary source of information was their doctors, in whom they placed great trust. Patients were compliant with treatments and rarely questioned them, although they sometimes failed to follow treatment recommendations. Zhang and Zhao^[39]^ included 136 COPD patients, dividing them randomly into an experimental group and a control group. The control group received a pulmonary rehabilitation program, while the experimental group participated in a pulmonary rehabilitation program coupled with an HBM-based intervention. The findings revealed that the HBM-based interventions reduced the levels of anxiety and depression in COPD patients, as well as the COPD Assessment Test scores, and also decreased levels of serum interleukin-6 and C-reactive protein in the experimental group. Wang et al^[40]^ focused on recruiting patients with moderate to severe COPD. Patients in the intervention group received a 20-to-30-minute HBM-based nursing intervention every 2 days after their condition stabilized during hospitalization, followed by a follow-up after discharge. Patients in the control group received standard care. A 3-month follow-up survey showed that the HBM-based nursing intervention enhanced patients’ health beliefs and self-efficacy regarding disease management, reduced dyspnea, improved exercise tolerance, and increased daily life activities.

In summary, the HBM is a psychologically driven theoretical framework that assesses individuals’ perceptions of their current health status, with a strong emphasis on the role of health knowledge. It enhances patients’ awareness of diseases and centers on beliefs to predict the likelihood of adopting health behavior changes. Beliefs are considered crucial for initiating and sustaining health actions. HBM focuses significantly on the beliefs formed through health education. Future research on this model could consider the impacts of demographic, socio-psychological, and structural factors on health education.

### 2.5. Theory of planned behavior

The TPB, proposed by Ajzen in 1991 as an extension of the Theory of Reasoned Action, stands out as a simple yet effective model for predicting social behaviors, offering improved explanations of the impact of individual internal factors on health behaviors.^[41]^ At its core, TPB posits that behavioral intention is the primary determinant of behavior, influenced by 3 main factors: attitudes toward the behavior (beliefs about the outcomes of the behavior and evaluations of these outcomes), subjective norms (motivation to comply with these expectations), and perceived behavioral control (an individual’s assessment of their ability to perform the behavior successfully). Notably, perceived behavioral control, a central component of TPB, directly affects whether an individual will engage in a specific behavior^[42]^ After continuous validation and refinement, TPB has evolved into an open and applicable model capable of effectively predicting and explaining individual behavioral decisions. It has been successfully applied to predict a variety of behaviors in areas such as blood donation,^[43]^ vaccination,^[44]^ telerehabilitation,^[45]^ cancer screening,^[46]^ nurses’ intention to leave their job,^[47]^ and intentions regarding injection treatments,^[48]^ demonstrating strong explanatory power.

A meta-analysis conducted by Gourlan et al^[49]^ revealed that interventions based on the TPB effectively promote physical activity. In a study by McGuckin et al,^[50]^ a survey using the TPB questionnaire among 23 patients with respiratory diseases (COPD and/or asthma) demonstrated that the TPB model could explain a significant variance, serving as an effective predictor of self-monitoring behavior intentions within the sample. The study also underscored the importance of subjective norms and perceived behavioral control in TPB for predicting intentions. Furthermore, Chevance et al^[51]^ investigated 119 COPD patients during a 5-week pulmonary rehabilitation period, exploring the impact of TPB variables and implicit attitudes on exercise and sedentary behaviors. They also examined the relationship between behavioral intentions, implicit attitudes, and physical activities postrehabilitation, finding an increase in perceived behavioral control and intentions toward physical activity, along with a more positive attitude toward rehabilitation exercises. Ricke et al^[52]^ measured TPB, disease, and demographic characteristics among 196 COPD patients, with follow-up measures of alliance taken 3 months later and adherence to exercise provided by physical therapists 12 months later. The analysis indicated that incorporating the concept of alliance into TPB is a promising innovation, aiding in understanding the exercise compliance of patients guided by healthcare professionals. The study offered specific recommendations for healthcare providers to gather information on patients’ attitudes, perceived behavioral control, and alliance to inform their guidance and further psychosocial interventions, effectively addressing the psychosocial determinants of adherence within the patient-coach relationship.

In summary, the TPB is among the most widely applied and successful behavior change theories in psychology. Originating from a psychological perspective, this theory analyzes individuals’ behavioral intentions, taking into account the influence of nonvolitional factors such as social norms, attitudes, and personal traits. TPB provides significant theoretical support for devising scientifically effective intervention strategies for exercise behavior.

## 3. Comparative analysis of the advantages and disadvantages of different theoretical models for health behaviors

Different models of behavior change each have their own advantages and disadvantages. The advantage of SDT lies in its strong emphasis on individual awareness, with motivation at its core. It starts from the psychological mechanisms generated by motivation to explain and predict the connections between different orientations and outcomes, effectively enhancing self-worth, perceived benefits from illness, and enthusiasm for self-management.^[21]^ However, the theory also has shortcomings, specifically that it overlooks the impact of environmental factors on individual behavior. It assumes that the psychological hallmark of individual behavior is the flexible control of interactions between oneself and the environment, thereby underestimating the influence of external factors.

The strengths of SCT include its richness in concepts and depth in theoretical exploration, providing a unified framework for content and interventions. However, a drawback of SCT is its complexity, which can complicate the design of practical programs due to the multitude of factors that need to be considered. This complexity often makes implementation challenging, and the theory does not allow for tailored guidance strategies based on the specific stages of the target population’s behavior change process.

The strengths of the TTM include its sensitivity to changes in each stage, allowing for targeted interventions and timely adjustments to intervention plans, which enhance patient engagement in health behaviors. However, it also has its shortcomings. The determination of which stage a patient is in can be subjective, and the division of stages may appear arbitrary. Moreover, there has not yet been a comparison of the outcomes between staged interventions and nonstaged interventions.

The advantage of HBM is that it attaches great importance to disease-related information support.

The HBM excels in its focus on disease-related informational support, significantly reinforcing patients’ health beliefs and showing noticeable effects on self-care capabilities. It effectively explains how informational and psychological factors influence individual health behaviors.^[37]^ However, HBM falls short as it does not account for other individual factors that influence behavior. It fails to fully explain how social environmental variables like policies and economic conditions affect individual health behaviors. Additionally, patients with poor compliance may form health beliefs but lack the energy and motivation to act on them.

The TPB excels at thoroughly explaining the process of individual behavioral decision-making. It offers flexibility and openness, which allows for the modification of its components to suit specific research needs, setting it apart from traditional theories. However, TPB has its shortcomings; it does not fully consider all influencing factors, notably overlooking individual cognition, affective, or emotional states. As a result, it falls short in its ability to predict whether an individual will actually engage in a specific behavior.

## 4. The guiding significance of theoretical models in pulmonary rehabilitation practice

As an integral component of scientific inquiry, theoretical models furnish a conceptual foundation for describing, explaining, and predicting phenomena, offering clarity in understanding relationships within systems and structuring research questions or constructing prior hypotheses. This article reviews the application status of various theoretical models in pulmonary rehabilitation research, emphasizing that regardless of the chosen model, pulmonary rehabilitation practice should focus on a central goal: improving health behaviors, and proceed from 2 main directions: healthcare providers and patients. Theoretical models should serve as pillars guiding practice, enhancing the integration of theory with practical exploration from diverse perspectives to establish comprehensive pulmonary rehabilitation management models and processes. This aims fundamentally to enhance the long-term adherence to health behaviors in COPD patients, thereby alleviating clinical symptoms, enhancing exercise tolerance, and improving quality of life. Currently, various theoretical models provide robust theoretical backing for the field of pulmonary rehabilitation, facilitating the effective bridging of theory and practice. They offer valuable insights for standardizing clinical management and enhancing the efficacy of nursing interventions and play a crucial role in aiding healthcare professionals in conducting accurate assessments and implementing personalized pulmonary rehabilitation strategies, meriting widespread clinical adoption.

## 5. Future directions and policy recommendations

### 5.1. Enhancing the influence of theoretical frameworks on pulmonary rehabilitation health behavior research

The application of theoretical frameworks to practical scenarios represents a pivotal step within the medical sector, anticipated to emerge as a key trend in forthcoming clinical investigations. Nonetheless, the current underutilization of theoretical constructs in research curtails the comprehensive understanding of relevant phenomena and curbs the implementation of adequate interventions. Investigations reveal that despite the presence of numerous mature and continuously optimized theories of behavioral change, a scant proportion of researchers employ these theories in devising interventions aimed at altering health behaviors within pulmonary rehabilitation contexts.^[18]^ A significant segment of the pulmonary rehabilitation research community has yet to acknowledge the crucial role of theoretical models in facilitating the education, intervention, and analysis of factors influencing patient health behavior modification. Consequently, prospective research directions should focus on elevating the recognition and application of theoretical tools among healthcare practitioners, advocating for an informed and efficacious amalgamation of theory with empirical research. This approach is instrumental in further substantiating and enhancing theoretical models, contributing to the discipline’s progression; amplifying research demographic scopes and endeavoring to amalgamate theoretical perspectives relevant to healthcare management with patient-centric interventions. Such efforts aim to formulate holistic pulmonary rehabilitation strategies, augmenting management efficiency and ensuring targeted interventions, thereby fostering the widespread adoption of diverse theoretical frameworks within China’s pulmonary rehabilitation milieu.

### 5.2. Dialectical selection of theoretical models

Various theoretical models exhibit unique characteristics in their content and application. For instance, SDT emphasizes the significance of individual initiative and intrinsic motivation in behavioral decision-making; SCT highlights the close relationship between behavior, environment, and the individual, with any 2 factors capable of mutually influencing each other; the TTM focuses on patient-centric interventions tailored to the specific stages of the patient, aiding in meeting their needs more effectively; the HBM underscores the pivotal role of individual beliefs in health behaviors, paying special attention to disease risk perceptions and cognitive beliefs; while the TPB posits that personal intent is the most direct influence on behavior, with attitudes, subjective norms, and perceived behavioral control also playing crucial roles. Currently, research in China’s field of pulmonary rehabilitation often directly employs theoretical models from abroad. Given the cultural and societal differences between domestic and international contexts, it is essential to further investigate the applicability and effectiveness of these models in the Chinese setting. Therefore, clinical nursing practitioners and researchers should conduct in-depth analyses based on specific issues, considering the applicability, scope, and methodologies of theoretical models. This approach involves the creative and dialectical selection of the most suitable theoretical model for different purposes.

### 5.3. Identifying and overcoming barriers to theoretical implementation

As an increasing number of researchers acknowledge the integration of theory with practice, theories play a crucial role in partially understanding complex realities and fostering behavioral changes toward positive health outcomes. However, significant challenges remain in effectively applying theories of behavioral change to facilitate individuals’ pulmonary rehabilitation health behavior transformation and future health planning. To address these challenges, it is imperative first to enhance healthcare professionals’ awareness and appreciation of behavioral change theories, recognizing their importance and necessity in the domain of pulmonary rehabilitation behavior. Nursing managers should strengthen training and motivational management to foster healthcare professionals’ initiative and critical thinking. Furthermore, specialized training and learning sessions should be conducted to encourage healthcare professionals to gain a deep understanding of various theories, master their methods and essence proficiently, and select appropriate theoretical perspectives for accurate theoretical guidance in research. This approach aims to achieve more effective and comprehensive pulmonary rehabilitation outcomes. Lastly, focusing on the differences in theoretical implementation and application effects across hospitals of varying levels and sizes in China is essential. Empirical studies conducted within diverse hospital cultural contexts should expand the theoretical framework, forming effective models for broader application.

### 5.4. Focusing on the development of new theoretical models

Health behavior change represents a long-term, complex, and multifaceted process involving a multitude of personal, societal, and environmental factors. To achieve optimal rehabilitation outcomes, interventions in pulmonary rehabilitation health behavior may require the integration of various theories and strategies. Existing theories of behavioral change appear somewhat outdated, lacking feedback on dynamic elements, failing to consider the instability and variability of factors, and not adequately addressing the integrative influence of social, economic, psychological, environmental, and personal cognition factors. Therefore, the development of new theoretical models, taking advantage of existing theories while compensating for their lack of dynamism and incorporating the strengths of traditional theories, is crucial. Constructing theoretical models that are more suitable for China’s cultural context and continually integrating research with new theoretical models represent a viable path for the evolution of the field of pulmonary rehabilitation health behavior. It also signifies an important improvement and refinement of traditional theories.

## 6. Conclusion

In the field of pulmonary rehabilitation, a multitude of theoretical models are being continuously validated and evolved through an increasing number of studies, offering fresh perspectives for addressing issues related to pulmonary rehabilitation health. Given the complexity of factors influencing patients’ engagement in pulmonary rehabilitation behaviors, the judicious selection and application of relevant theoretical models are crucial. By investigating the factors that affect COPD patients’ participation in pulmonary rehabilitation behaviors and identifying controllable elements, effective strategies can be devised. Consequently, focusing on the development of new theoretical models and broadening their application in the domain of pulmonary rehabilitation health behaviors not only enriches the diversity of theories but also provides a solid theoretical foundation for establishing scientific and reliable management plans.

## Author contributions

**Conceptualization:** Yuyin Chen, Xiuhong Long.

**Data curation:** Yuyin Chen, Ruyi Tan, Huiqiong Tu.

**Formal analysis:** Yuyin Chen, Ruyi Tan, Huiqiong Tu.

**Writing—original draft:** Yuyin Chen, Ruyi Tan, Huiqiong Tu.

**Writing—review & editing:** Yuyin Chen, Ruyi Tan, Xiuhong Long, Huiqiong Tu.

## References

[1] Venkatesan P. Gold COPD report: 2024 update. Lancet Respir Med. 2024;12:15–6. 38061380 10.1016/S2213-2600(23)00461-7

[2] Kahnert K Jörres RA Behr J Welte T. The diagnosis and treatment of COPD and its comorbidities. Dtsch Arztebl Int. 2023;120:434–44. 36794439 10.3238/arztebl.m2023.027 PMC10478768

[3] Fabbri LM Celli BR Agustí A. COPD and multimorbidity: recognising and addressing a syndemic occurrence. Lancet Respir Med. 2023;11:1020–34. 37696283 10.1016/S2213-2600(23)00261-8

[4] Safiri S Carson-Chahhoud K Noori M. Burden of chronic obstructive pulmonary disease and its attributable risk factors in 204 countries and territories, 1990-2019: results from the global burden of disease study 2019. BMJ. 2022;378:e069679. 35896191 10.1136/bmj-2021-069679 PMC9326843

[5] Wang C Xu J Yang L. China Pulmonary Health Study Group. Prevalence and risk factors of chronic obstructive pulmonary disease in China (The China Pulmonary Health [CPH] study): a national cross-sectional study. Lancet. 2018;391:1706–17. 29650248 10.1016/S0140-6736(18)30841-9

[6] Li RT Rao ZZ Fu YH. Prediction on the burden of disease of chronic obstructive pulmonary disease and simulation of the effectiveness of controlling risk factors in China by 2030. Zhonghua Liu Xing Bing Xue Za Zhi. 2022;43:201–6. 35184485 10.3760/cma.j.cn112338-20210803-00606

[7] López-Campos JL Tan W Soriano JB. Global burden of COPD. Respirology. 2016;21:14–23. 26494423 10.1111/resp.12660

[8] Ko FW Chan KP Hui DS. Acute exacerbation of COPD. Respirology. 2016;21:1152–65. 27028990 10.1111/resp.12780 PMC7169165

[9] Troosters T Janssens W Demeyer H Rabinovich RA. Pulmonary rehabilitation and physical interventions. Eur Respir Rev. 2023;32:220222. 37286219 10.1183/16000617.0222-2022 PMC10245142

[10] Janssens W Verleden GM. Nonpharmacological interventions in COPD. Eur Respir Rev. 2023;32:230028. 36948503 10.1183/16000617.0028-2023 PMC10032612

[11] Spruit MA Singh SJ Garvey C. ATS/ERS Task Force on Pulmonary Rehabilitation. An official American Thoracic Society/European Respiratory Society statement: key concepts and advances in pulmonary rehabilitation. Am J Respir Crit Care Med. 2013;188:e13–64. 24127811 10.1164/rccm.201309-1634ST

[12] Rochester CL Vogiatzis I Holland AE. ATS/ERS Task Force on Policy in Pulmonary Rehabilitation. An official American Thoracic Society/European Respiratory Society policy statement: enhancing implementation, use, and delivery of pulmonary rehabilitation. Am J Respir Crit Care Med. 2015;192:1373–86. 26623686 10.1164/rccm.201510-1966ST

[13] Holland AE Cox NS Houchen-Wolloff L. Defining modern pulmonary rehabilitation. An official American Thoracic Society workshop report. Ann Am Thorac Soc. 2021;18:e12–29. 33929307 10.1513/AnnalsATS.202102-146ST PMC8086532

[14] Choi JY Ryu EJ Jin X. Effect of self-management education using pictogram-based content of health information on outcomes in Korean patients with chronic obstructive pulmonary disease: a randomized controlled trial. Geriatr Nurs. 2023;54:324–30. 37948887 10.1016/j.gerinurse.2023.10.030

[15] Chan SC Patrick Engksan J Jeevajothi Nathan J. RESPIRE Collaboration. Developing a home-based pulmonary rehabilitation programme for patients with chronic respiratory diseases in Malaysia: a mixed-method feasibility study. J Glob Health. 2023;13:04099. 37883199 10.7189/jogh.13.04099 PMC10602205

[16] Hanlon P Daines L Campbell C McKinstry B Weller D Pinnock H. Telehealth interventions to support self-management of long-term conditions: a systematic metareview of diabetes, heart failure, asthma, chronic obstructive pulmonary disease, and cancer. J Med Internet Res. 2017;19:e172. 28526671 10.2196/jmir.6688 PMC5451641

[17] Redfern J Singleton AC Raeside R. Integrated text messaging (ITM) for people attending cardiac and pulmonary rehabilitation: a multicentre randomised controlled trial. Ann Phys Rehabil Med. 2023;67:101800. 38118248 10.1016/j.rehab.2023.101800

[18] Coll F Cavalheri V Gucciardi DF Wulff S Hill K. In people with COPD, there is limited evidence that exercise training reduces sedentary time, and behavior change techniques are poorly reported: systematic review and meta-analysis. Phys Ther. 2021;101:pzab097. 33742675 10.1093/ptj/pzab097

[19] McCullough AR Ryan C Macindoe C. Behavior change theory, content and delivery of interventions to enhance adherence in chronic respiratory disease: a systematic review. Respir Med. 2016;116:78–84. 27296825 10.1016/j.rmed.2016.05.021

[20] Flannery M. Self-determination theory: intrinsic motivation and behavioral change. Oncol Nurs Forum. 2017;44:155–6. 28222078 10.1188/17.ONF.155-156

[21] Van Lange PA Higgins ET Kruglanski AW. Handbook of Theories of Social Psychology. SAGE Publications; Thousand Oaks, California, USA. 2011:1–1144.

[22] Ntoumanis N Ng JYY Prestwich A. A meta-analysis of self-determination theory-informed intervention studies in the health domain: effects on motivation, health behavior, physical, and psychological health. Health Psychol Rev. 2021;15:214–44. 31983293 10.1080/17437199.2020.1718529

[23] Drennan VM Ross F. Global nurse shortages—the facts, the impact and action for change. Br Med Bull. 2019;130:25–37. 31086957 10.1093/bmb/ldz014

[24] Knox L Norris G Lewis K Rahman R. Using self-determination theory to predict self-management and HRQoL in moderate-to-severe COPD. Health Psychol Behav Med. 2021;9:527–46. 34150367 10.1080/21642850.2021.1938073 PMC8189057

[25] Bandura A. Social cognitive theory: an agentic perspective. Annu Rev Psychol. 2001;52:1–26. 11148297 10.1146/annurev.psych.52.1.1

[26] Borah P Lorenzano K Yel E Austin E. Social cognitive theory and willingness to perform recommended health behavior: the moderating role of misperceptions. J Health Commun. 2024;29:49–60. 37970863 10.1080/10810730.2023.2282035

[27] Cook DA Artino AR Jr. Motivation to learn: an overview of contemporary theories. Med Educ. 2016;50:997–1014. 27628718 10.1111/medu.13074 PMC5113774

[28] Jolly K Sidhu MS Hewitt CA. Self management of patients with mild COPD in primary care: randomised controlled trial. BMJ. 2018;361:k2241. 29899047 10.1136/bmj.k2241 PMC5998171

[29] Aliakbari F Alipour FM Tavassoli E Sedehi M. The effect of empowerment program based on the social cognitive theory on the activity of daily living in patients with chronic obstructive pulmonary disease. J Educ Health Promot. 2020;9:146. 32766331 10.4103/jehp.jehp_752_19 PMC7377143

[30] Kosteli MC Heneghan NR Roskell C. Barriers and enablers of physical activity engagement for patients with COPD in primary care. Int J Chron Obstruct Pulmon Dis. 2017;12:1019–31. 28405162 10.2147/COPD.S119806 PMC5378459

[31] Prochaska JO Velicer WF. The transtheoretical model of health behavior change. Am J Health Promot. 1997;12:38–48. 10170434 10.4278/0890-1171-12.1.38

[32] Marcus BH Simkin LR. The transtheoretical model: applications to exercise behavior. Med Sci Sports Exerc. 1994;26:1400–4. 7837962

[33] Chen X Yang X-Y Liu S-M. Effects of rehabilitation exercise intervention on the pulmonary function and quality of life in patients with chronic obstructive pulmonary disease based on trans-theoretical model. Chin Gen Pract. 2018;21:3240.

[34] Hellem E Bruusgaard KA Bergland A. Exercise maintenance: COPD patients’ perception and perspectives on elements of success in sustaining long-term exercise. Physiother Theory Pract. 2012;28:206–20. 21823993 10.3109/09593985.2011.587502

[35] Rosenstock IM Strecher VJ Becker MH. Social learning theory and the health belief model. Health Educ Q. 1988;15:175–83. 3378902 10.1177/109019818801500203

[36] Jones CJ Smith H Llewellyn C. Evaluating the effectiveness of health belief model interventions in improving adherence: a systematic review. Health Psychol Rev. 2014;8:253–69. 25053213 10.1080/17437199.2013.802623

[37] Khodaveisi M Azizpour B Jadidi A Mohammadi Y. Education based on the health belief model to improve the level of physical activity. Phys Act Nutr. 2021;25:17–23. 35152620 10.20463/pan.2021.0022 PMC8843841

[38] Simon J. Attitudes of Hungarian asthmatic and COPD patients affecting disease control: empirical research based on health belief model. Front Pharmacol. 2013;4:135. 24312052 10.3389/fphar.2013.00135 PMC3826076

[39] Zhang Y Zhao X. Effects of the health belief model-based intervention on anxiety, depression, and quality of life in chronic obstructive pulmonary disease. Neuroimmunomodulation. 2021;28:129–36. 34062535 10.1159/000512993

[40] Wang Y Zang XY Bai J Liu S-Y Zhao Y Zhang Q. Effect of a health belief model-based nursing intervention on Chinese patients with moderate to severe chronic obstructive pulmonary disease: a randomised controlled trial. J Clin Nurs. 2014;23:1342–53. 24102822 10.1111/jocn.12394

[41] Bosnjak M Ajzen I Schmidt P. The theory of planned behavior: selected recent advances and applications. Eur J Psychol. 2020;16:352–6. 33680187 10.5964/ejop.v16i3.3107 PMC7909498

[42] Sur MH Jung J Shapiro DR. Theory of planned behavior to promote physical activity of adults with physical disabilities: meta-analytic structural equation modeling. Disabil Health J. 2022;15:101199. 34456174 10.1016/j.dhjo.2021.101199

[43] Siu JY Chan EA Li AS Lee YM. Motivations and deterrents of blood donation among blood donors during the COVID-19 pandemic in Hong Kong. Health Expect. 2022;25:3192–201. 36245309 10.1111/hex.13626 PMC9700176

[44] Antonopoulou V Goffe L Meyer CJ. A comparison of seasonal influenza and novel covid-19 vaccine intentions: a cross-sectional survey of vaccine hesitant adults in England during the 2020 pandemic. Hum Vaccin Immunother. 2022;18:2085461. 35816683 10.1080/21645515.2022.2085461 PMC9621000

[45] Wang MY Chen H Gong C. Understanding the use intention and influencing factors of telerehabilitation in people with rehabilitation needs: a cross-sectional survey. Front Public Health. 2023;11:1274080. 38026371 10.3389/fpubh.2023.1274080 PMC10654628

[46] Sun Y Yuan J Liu W. Predicting rural women’s breast cancer screening intention in China: a PLS-SEM approach based on the theory of planned behavior. Front Public Health. 2022;10:858788. 35480590 10.3389/fpubh.2022.858788 PMC9035887

[47] Zhang F Lin C Li X. The relationships between burnout, general wellbeing, and psychological detachment with turnover intention in Chinese nurses: a cross-sectional study. Front Public Health. 2023;11:1216810. 37546331 10.3389/fpubh.2023.1216810 PMC10399590

[48] Hsu SH Tang KP Lin CH Chen P-C Wang L-H. Applying the theory of planned behavior to investigate type 2 diabetes patients’ intention to receive injection therapy. Front Public Health. 2023;11:1066633. 36875423 10.3389/fpubh.2023.1066633 PMC9978190

[49] Gourlan M Bernard P Bortolon C. Efficacy of theory-based interventions to promote physical activity. A meta-analysis of randomised controlled trials. Health Psychol Rev. 2016;10:50–66. 25402606 10.1080/17437199.2014.981777

[50] McGuckin C Prentice GR McLaughlin CG Harkin E. Prediction of self-monitoring compliance: application of the theory of planned behaviour to chronic illness sufferers. Psychol Health Med. 2012;17:478–87. 22111866 10.1080/13548506.2011.630399

[51] Chevance G Héraud N Varray A Boiché J. Change in explicit and implicit motivation toward physical activity and sedentary behavior in pulmonary rehabilitation and associations with postrehabilitation behaviors. Rehabil Psychol. 2017;62:119–29. 28414502 10.1037/rep0000137

[52] Ricke E Dijkstra A. Determinants of exercise adherence in a prospective cohort study of patients with chronic obstructive pulmonary disease: an application of the theory of planned behavior and the concept of alliance. Med Res Arch. 2023;11.

